# A Longitudinal Study of Physical and Mental Health and Health-Related Attitudes Among Music Students: Potentials and Challenges for University Health Promotion Programs

**DOI:** 10.3389/fpsyg.2022.885739

**Published:** 2022-07-04

**Authors:** Magdalena Rosset, Eva Baumann, Eckart Altenmüller

**Affiliations:** ^1^Department of Journalism and Communication Research, Hanover University of Music, Drama and Media, Hanover, Germany; ^2^Institute of Music Physiology and Musicians’ Medicine, Hanover University of Music, Drama and Media, Hanover, Germany

**Keywords:** musicians’ health, playing-related pain, mental health, performance studies, music education studies, music students, prevention

## Abstract

**Objective:**

Well-being of music students has been an increasing matter of concern since studies show that up to 50% of beginners suffer from playing-related pain or anxiety. The aim of this longitudinal study was to examine health status, health-related attitudes, behaviors, knowledge, skills, and coping strategies of students at the beginning of their education at a music university and at the end of their second semester.

**Methods:**

Based on a longitudinal online survey conducted among students at a German music university since 2017, we investigated mental and physical health status, health-related attitudes, knowledge, skills, behaviors, and coping strategies of music students at the beginning of their first year (*n* = 205). We analyzed differences between performance and music education majors and between students playing different main instruments. In a subsample (*n* = 62), we additionally analyzed changes between the beginning of the music students’ first and the end of their second semester, also depending on whether they attended courses on musicians’ health.

**Results:**

Music students are already in demand when they enter a music university, practicing on average almost 3 h daily. Compared to other body regions, pain in shoulders/back is most prevalent in first-year students, especially in those playing string instruments. Performance majors reported better knowledge about health risks and protective measures for musicians, better coping abilities, and practiced more than music education majors. First-year students assessed their overall and mental health status at the beginning of their first semester mainly as good, but we found a decrease in mental health status at the end of the second semester. After two semesters, students attending courses on musicians’ health showed increased knowledge and skills regarding different aspects of musicians’ health.

**Conclusion:**

The health status of music students when they first enter a music university is still a concern. Information and practical courses enabling students to prevent overuse and cope with performing anxiety and other stressors are important components of a comprehensive study program. Knowledge about music students’ needs can help conservatories better respond to the requirements and develop courses and measures supporting students from the beginning of their education.

## Introduction

The well-being of music students has been a longstanding matter of concern, since studies show that up to 50% of beginners suffer from playing-related pain or anxiety, mostly caused by stress and overuse (e.g., Fry, 1987; Lockwood, 1988; Zaza, 1992; Guptill et al., 2000). Generally, music students identify particularly with their choice of profession (Spahn et al., 2004) and have to deal with particular risk factors linked to their specific situation: (1) students start their professional training in childhood and adolescence, (2) playing music is linked to pleasure, strong emotions, and identity, (3) students frequently work at their physical and mental limits, (4) performing on stage in front of audiences, fellow students, and peers involves high societal pressure and frequently stress and anxiety, (5) the design of musical instruments is in many instances historical and implies unfavorable ergonomics (Spahn, 2006; Altenmüller and Jabusch, 2012).

Based on the specific challenges of studying music, it is not surprising that music students frequently suffer from medical conditions such as pain (Spahn et al., 2004; Williamon and Thompson, 2006; Spahn and Möller, 2011), performance anxiety (Williamon and Thompson, 2006; Spahn, 2011), and psychological problems, such as low self-esteem, dysfunctional perfectionism, and negative self-concept (Puls, 2004; Spahn et al., 2004; Zander et al., 2010). For instance, studies revealed that music students showed worse general health and worse physical health than amateur musicians (Antonini Philippe et al., 2019) and worse general health than university students of other disciplines (Spahn et al., 2004; Araújo et al., 2017).

Studying music involves rigorous daily practicing hours, and as a specific goal of the studies, performing under evaluative contexts, for example, during master classes and concerts. Music university education, therefore, needs to implement courses in order to prepare for auditions, public appearances, and other stressful evaluative situations. These may include virtual reality training of performances (Williamon et al., 2014), however, the basis is a comprehensive prevention program addressing bodily as well as psychological health, establishing awareness for health-related aspects, and impart practical knowledge concerning preventive health behavior.

Indeed, such preventive programs have been officially recommended in several countries, e.g., Germany (Spahn, 2006), the United States (Chesky et al., 2006), and the United Kingdom (e.g., Williamon and Thompson, 2006; Clark et al., 2013), and were systematically evaluated in some places, however, frequently with a small number of participants only. Spahn et al. (2001) investigated health outcomes following a weekly course of theoretic information about health-related behaviors and practical exercises in 22 music students and 22 controls. They showed improvements of playing-related pain symptoms, general symptom frequency, and emotional disturbances and anxiety levels. In a study by López and Martínez (2013) a course, focusing on health promotion and education about common medical problems, as well as on advice regarding posture, warm-up strategies, and effective prevention strategies was offered. Participants in the experimental group (*n* = 90) improved their body awareness by 91% and their injury rate decreased by 78%. Zander et al. (2010) demonstrated positive changes both for physical and psychological well-being after a health promotion course, however, in this study no control group was included. Generally, although sample sizes are mostly small (under 30), health prevention courses promoting healthy habits yield positive effects in many countries, e.g., the United States (Barton and Feinberg, 2008), South Africa (Panebianco-Warrens et al., 2015), or Iceland (Árnason et al., 2018).

In a study including 246 performance students from Manchester and London conservatories, Kreutz et al. (2008) focused on the musculoskeletal and nonmusculoskeletal health problems. They identified associations between health problems and behaviors and analyzed their relation to musical practice and performance. A high prevalence of musculoskeletal and nonmusculoskeletal problems was significantly impacting the perceived quality of practice. The authors conclude that the quality of musical practice and performance is threatened by a combination of problems specific to the upper extremities and spine as well as fatigue. In consequence, they propose the implementation of programs emphasizing the importance of physical fitness generally, paying particular attention to posture and the upper limbs, and focusing on the prevention of fatigue.

At the Hanover University of Music, Drama and Media, Germany, we have established and further developed such a program, beginning in 1994. First, a lecture entitled “The bodily and mental basis of healthy musicianship” was implemented, informing students about health issues and preventive strategies. This was supplemented by a musicians’ clinic enabling students short-term, low-threshold access to a Performing Arts Medicine Specialist (author EA). In the late nineties, individual lessons in Feldenkrais and Alexander-technique were installed. From 2000 on, tutorials in small groups informing about preventive strategies and techniques such as performance training in order to overcome performance anxiety were additionally offered. In 2002, a survey evaluated these programs and assessed the health status of the university’s music students (Gräser, 2004). The results showed that a majority of students suffered from playing-related pain. Furthermore, most respondents rated the health-related offers as important or even very important (Gräser, 2004). The survey also found that those students who reported higher burdens, i.e., more and longer practice times, worse physical and mental health status, more frequent discomfort, were more likely to attend preventive courses at the university (Gräser, 2004). Thus, higher physical and mental demands lead to greater willingness to engage in programs regarding musicians’ health. Integrating compulsory health classes in the curriculum can be a means to raise awareness, increase knowledge and skills regarding different aspects of musicians’ health, and encourage music students to take preventive measures.

In such lectures and courses, music students can further be taught to rely on resources that are capable of positively influencing health and well-being, for instance, coping strategies, the individual importance that is ascribed to health, or striving to maintain a healthy lifestyle (Perkins et al., 2017). Here, the present Hanover program differs from programs established in other music universities in Germany and elsewhere (Grieco et al., 1989; Zander et al., 2010; Spahn et al., 2014; Matei and Ginsborg, 2021), since it is considerably more comprehensive. First, lectures and tutorials are compulsory courses for all bachelor students in performance and music education; however, students are free to take these required courses in any semester of their education. Furthermore, written exams and regular evaluations of the faculty assure quality of teaching. Second, it involves a peer-learning approach for the tutorials. Performance master students tutor bachelor students in small groups for 2 h weekly emphasizing the benefits of self-awareness, a healthy lifestyle, and avoidance of a no pain, no gain attitude. Thirdly, we institutionalized each semester small group practical courses (“laboratories”) in an interdisciplinary atmosphere for 2 h twice a week, including bachelor and master students of all study programs (jazz, popular music, classical), focusing on “how to practice” and “how to overcome performance anxiety.” For the former, each week, a student presents a “technical” problem, e.g., a particularly difficult passage, or high demands for endurance, speed, coordination etc., which then is discussed, and suggestions for solutions are presented. For the latter, students perform in front of each other under the guidance of a psychotherapist every week, with preliminary bodily and mental exercises and subsequent supervised performance evaluation. Finally, we offer a free, low-threshold face-to-face counseling service under medical confidentiality conditions by a MD, experienced in musician’s medicine, neurology, and psychiatry (author EA) and a registered psychotherapist specialized for treatment of musicians, addressing health concerns, health behaviors, and general worries concerning the course of the studies, anxieties, and conflicts. Here, frequent issues are relationship problems, loneliness, professional pressures, and specific topics, such as usage of beta blockers to overcome performance anxiety, etc. During the coronavirus pandemic (which is not addressed in our study since the data have been collected before the pandemic), the latter was of vital importance for the well-being of students (Rosset et al., 2021).

### Research Objectives

While the above mentioned research groups investigated health status of music students, thereby partially also taking a longitudinal perspective, the difference to our study lays in the sample size and most importantly in the quality of the intervention. A specific feature of our program is that courses are obligatory for performance and music education students and that they comprise peer-learning, “laboratories,” and individual counseling. The present study adds to the current state of research by investigating at the same time not only the health status of music students when they first enter a music university but also health-related attitudes, behaviors, knowledge, and skills as well as their coping strategies and the development of these aspects over the course of the first two semesters as well as the impact of a comprehensive health prevention program.

Knowledge about the students’ health status when they first enter a music university is critical to best address their needs early on in their music education. Additionally, we add a longitudinal perspective by examining how the health status evolves over the course of the music students’ first year and what role attending courses on musicians’ health plays. Analyzing the impact of the high demands of studying music and identifying vulnerable groups among first-year students can contribute to improving the preventive programs at music universities and help to address students in need of specific interventions.

The first aim of this study was to examine the health status as well as health-related aspects of music students when they first enter a music university. Therefore, our first research question is:

*RQ1*: What are physical and mental health status, health-related attitudes, knowledge, skills, and behaviors, and coping strategies of music students at the beginning of their music university education?

The curriculum and requirements of performance classes differ from those of music education programs. Usually, performance majors concentrate on instrumental practice, supplemented with ear-training courses, music history, and orchestra and ensemble training. In contrast, music education majors practice their main instrument less and are more involved in the multi-instrumental practice, including singing, choir conducting, harmony classes, music history classes, improvisation, and theoretical pedagogical and research seminars. Therefore, students deciding to enroll in either of the programs may differ already before entering a music university, e.g., in their level of performance anxiety (e.g., Nawrocka et al., 2014):

*H1*: Performance majors and music education majors differ regarding the aspects under investigation at the beginning of their studies.

We further focus on differences between instrument groups, since different instruments require a different amount of practice and challenge the body in different ways (e.g., Jørgensen, 2002):

*H2*: Students playing different main instruments differ regarding the aspects under investigation at the beginning of their studies.

Since a further aim of our study is to explore the development of the health status after two semesters of training at a music university and the effect of courses on musicians’ health, we include a longitudinal perspective. The second research question and the third hypothesis are concerned with the development of the aspects under investigation over the course of the first two semesters at a music university and differences depending on whether students attended courses on musicians’ health. The hypothesis is founded in the results of the previous longitudinal studies reported above.

*RQ2*: How do the aspects under investigation develop over the course of the first two semesters at a music university for students attending and not attending courses on musicians’ health?

*H3*: After two semesters at a music university, students attending courses on musicians’ health show an improved health status and improved health-related attitudes, knowledge, and skills compared to students not taking courses on musicians’ health.

## Materials and Methods

Since 2017, each year in the first month of the semester, an online survey was distributed *via* e-mail to all first-year bachelor students enrolled in performance training and music education training at a German music university. Performance training comprises the programs musical performance (classical), pianoforte, jazz and jazz-related music, and popular music. Music education training comprises the programs music performance and education, and an interdisciplinary bachelor’s degree.

Additionally, we conducted follow-up studies at the end of each academic year to gain insights into the development of health status, health-related attitudes, and behaviors in the same cohorts. These follow-up studies were always conducted in the last month of the second semester. Since we wanted to capture the health status of first-year students in regular times and the coronavirus pandemic had intense effects and posed specific challenges for studying music that needs to be investigated in detail and, thereby, take specific determinants of the pandemic into account (see Rosset et al., 2021), we decided only to include the cohorts that started their music education in 2017, 2018, and 2019. For the longitudinal perspective, we included the follow-up surveys for the students that took up their studies in 2017 and 2018 and were surveyed again in 2018 and 2019, respectively. Correspondingly, for the longitudinal perspective, we excluded the first-year students who began their music studies in 2019 and were surveyed again in 2020 and we excluded the first-year students who began studying in 2020 and 2021 altogether.

### Participants

The overall sample consisted of *n* = 205 first-year students: The first survey in 2017 was answered by 75 first-year students (response rate: 62%), the second in 2018 by 86 (response rate: 69%), and the third in 2019 by 44 (response rate: 35%). Of the overall 161 first-year students in 2017 and 2018, 62 answered the follow-up survey at the end of their second semester (2017: *n* = 27, 2018: *n* = 35). The longitudinal analyses were limited to the *n* = 62 students who completed both surveys at the beginning of their first semester and at the end of their second semester.

The survey was available in German and English, with 181 first-year respondents (88%) choosing the German version and 24 respondents (12%) the English version. In the follow-up survey, 58 respondents (94%) chose the German version and 4 (7%) the English version.

### Procedure

The students were recruited *via* a mailing list including all first-year students. At the beginning of the online survey, the subject and purpose of the study and the voluntary nature of participation were explained, the anonymity and confidential handling of the data was assured, and the participants were informed that they could withdraw their consent to participate in the survey at any time. The participants gave their informed consent to take part in the study prior to entering the main survey. The study was approved by the joint ethics committee of the Leibniz University Hannover and the Hanover University of Music, Drama and Media (EV-LUH 9/2017). We furthermore adhere to the ethics regulations of our university according to the guidelines of the German Research Foundation and the Declaration of Helsinki. Participants were compensated for their time with 20 Euro.

### Measures

The same measurements were used in all waves of the survey. Besides *gender*, *age*, *program of study*, *first citizenship*, and *main instrument* (which was later grouped into wind, keyboard, string, plucking, and percussion instruments, as well as voice and theoretical programs), the respondents were asked to self-assess their *overall health status* (“How would you describe your overall health?”) on a five-point Likert-type scale (1 “very good” to 5 “very bad”). Respondents were also asked to indicate on a five-point Likert-type scale (1 “none at all” to 5 “very much”) their perceived stress over the past week in eight different domains (e.g., “feeling fearful”) to assess their *mental health status*. The items were taken from the eight-item symptom checklist (SCL-8), a short form of the SCL-25 measuring symptoms of depression and anxiety (Tambs and Røysamb, 2014). The scale showed high internal consistency in this study (first-year only: *α* = 0.86; beginning of first semester and end of second semester: *α* = 0.88) and the items were combined into a mean index with lower scores indicating better mental health status and higher scores indicating worse mental health status.

Based on frequency (0 “never,” 1 “once every 6 months or fewer,” 2 “more than once every 6 month, but not every month,” 3 “monthly,” 4 “more than once a month,” 5 “constantly”) and severity of *pain* (on a sliding scale from 0 “no pain at all” to 100 “very intense pain”) in back and shoulders, arms and hands, mouth and jaw, and hearing, a composite pain score for each of the body regions was derived by multiplying the intensity of pain and the frequency of pain and dividing the product by ten (a similar score is used in the Pain Frequency-Severity-Duration Scale, PFSD, Salamon et al., 2014). The composite score can range from 0 to 50. Additionally, the respondents were asked to assess their playing-related impairments due to pain (“When you add all your pain together, how strongly does it affect you when you play music?”) on a five-point scale (1 “not at all” to 5 “very strongly”). Additionally, the survey asked for *average daily practicing hours*.

The respondents were further asked to assess their perception of the *importance of health overall* (“How important is the general topic of health for you personally?”) and of *health particularly for musicians* (“In your opinion, how important is health for musicians?”) on a five-point scale (1 “not at all important” to 5 “very important”).

Based on the scale from Dutta-Bergman (2004), *health consciousness* was measured with five items (e.g., “I actively try to prevent disease and illness,” “Living life in the best possible health is very important to me.”) on a five-point Likert-type scale (1 “strongly disagree” to 5 “strongly agree”). The scale showed sufficient internal consistency (first-year only: *α* = 0.63; beginning of first semester and end of second semester: *α* = 0.66) and the items were combined into a mean index.

The questionnaire further asked about the perceived *level of knowledge about health risks for musicians* (“How well-informed to do you feel about the various health risks associated with the occupation of being a musician?”) and about *health protective measures for musicians* (“How well-informed do you feel about various methods of maintaining good personal health as a musician?”) on a five-point Likert-type scale (1 “not at all” to 5 “very well”).

Further, eight items asked about the *perceived level of knowledge and skills about different aspects of musicians’ health* (relaxation methods, stress and time management, mental practice and memorization, body posture and movement, e.g., “How well-informed/competent do you feel about proper body posture and movement while singing/playing?”). All items were measured on a five-point Liker-type scale (1 “not at all” to 5 “very well”) and combined into a mean index (first-year only: *α* = 0.74; beginning of first semester and end of second semester: *α* = 0.73).

*Performance anxiety* (“Performing situations make me nervous,” “Performing situations make me feel uneasy,” “Performing situations make me feel scared.”) and *coping with performance anxiety* (“I feel capable of dealing with my nervousness in performing situations,” “I feel capable of dealing with my uneasiness in performing situations,” “I feel capable of dealing with my fear in performing situations.”) were measured with three items, respectively, on a five-point Likert-type scale (1 “does not apply at all” to 5 “applies completely”). Both scales showed high internal consistency (first-year only: performance anxiety: *α* = 0.82, coping with performance anxiety: *α* = 0.89; beginning of first semester and end of second semester: performance anxiety: *α* = 0.78, coping with performance anxiety: *α* = 0.88) and the items were combined into a mean index for performance anxiety and for coping with performance anxiety, respectively.

Using the Stress and Coping Inventory (SCI; Satow, 2012), items regarding five different *coping strategies* were included (*social support*: e.g., “When I am stressed or under pressure, I find support from my partner or a good friend.”; *positive thinking*: e.g., “I tell myself that stress and pressure also have positive effects”; *active coping*: e.g., “I do everything to prevent stress in the first place.”; *faith*: e.g., “When I am stressed or under pressure, I find relief in my faith.”; *alcohol and cigarettes*: e.g., “When I am stressed or under pressure I relax with a glass of wine or beer in the evening.”). All five coping strategies were assessed with four items using a five-point Likert-type scale (1 “does not apply at all” to 5 “applies completely”). The five subscales showed sufficient internal consistencies (first-year only: social support: *α* = 0.76, positive thinking: *α* = 0.62, active coping: *α* = 0.73, faith: *α* = 0.77, alcohol and cigarettes: *α* = 0.77; beginning of first semester and end of second semester: social support: *α* = 0.82, positive thinking: *α* = 0.66, active coping: *α* = 0.74, faith: *α* = 0.82, alcohol and cigarettes: *α* = 0.74) and the items for each coping strategy were combined into a mean index.

In the follow-up survey, we further measured if the students had attended any courses on musicians’ health in their first year. We separately assessed if they attended the lecture “the bodily and mental basis of healthy musicianship,” the tutorial, or “laboratories” and combined the answers in one dichotomous variable indicating attendance in at least one course on musicians’ health.

### Data Analysis

All analyses were performed using SPSS (v. 28, Armonk, NY: IBM Corp.). Besides descriptive analyses of the sample using frequencies and percentages for categorical data and means (*M*) and standard deviations (*SD*) for numeric data, differences between performance majors and music education majors (H1), and between instrument groups (H2) were assessed using chi-square tests and multi-factor analyses of variance (ANOVAs) testing differences between majors and instrument groups in the same model, adjusting for gender, first citizenship, and cohort to control for the data collection in different years. We used G*Power (Faul et al., 2007) to determine the minimum effect sizes that could have been reliably detected based on our given sample size, *α* = 0.05, and a desired power of 0.8 (sensitivity analysis). Based on these values, the minimum detectable effect size to determine differences between majors was *f* = 0.20 (equals approximately $\eta_{p}^{2}$ = 0.04) and *f* = 0.26 (equals approximately $\eta_{p}^{2}$ = 0.06) to assess differences between students playing different main instruments.

To analyze differences between the beginning of the first semester and the end of the second semester (RQ2), we conducted repeated measures ANOVAs. For the repeated measures ANOVAs, sensitivity analysis with our given sample size of 62, *α* = 0.05, and a desired power of 0.80 gives a minimum detectable effect size of *f* = 0.18 (equals approximately $\eta_{p}^{2}$ = 0.03). To analyze the interaction between time and group (H3), we conducted mixed ANOVAs. As dependent variables we used the aspects under investigation, as the within-subjects factor we used time (beginning of first semester vs. end of second semester at a music university), and as between-subjects factor we used the group (attending courses on musicians’ health vs. not attending courses). For the mixed ANOVAs, sensitivity analysis showed a minimum detectable effect size of *f* = 0.18 (equals approximately $\eta_{p}^{2}$ = 0.03). In case of statistically significant interaction effects, simple main effects for both factors (using repeated measures ANOVA with separate groups for the within-subjects factor and one-way ANOVA for between-subjects factor) were determined.

## Results

### Sample Characteristics

Of the overall sample of first-year students, 35% (*n* = 71) of the respondents were performance majors and 65% (*n* = 134) were music education majors (see Table 1). There were slightly more female students in the sample. Students in the sample were on average 20.02 years old and most had a German first citizenship. In the sample, 25% of the students played wind instruments as main instrument, 22% played keyboard instruments, 22% played string instruments, 13% sang, 8% played plucked instruments, 5% played percussion instruments, and 4% of the students were enrolled in composition or music theory.

**Table 1:** Sample characteristics (performance majors and music education majors).

	Major						Total (*n* = 205)
Performance (*n* = 71, 35%)			Music education (*n* = 134, 65%)		
	n/M	%/SD	95% CI	n/M	%/SD	95% CI	n/M	%/SD	95% CI
Gender^***^									
Female	26	38%		88	66%		114	56%	
Male	43	62%		46	34%		89	43%	
Age	19.70	2.41	[19.13, 20.27]	20.19	2.63	[19.74, 20.64]	20.02	2.56	[19.67, 20.38]
First citizenship^***^									
German	47	67%		119	89%		166	81%	
Other	23	33%		15	11%		38	19%	
Main instrument									
Wind instruments	19	27%		32	24%		51	25%	
Keyboard instruments	9	13%		37	28%		46	22%	
String instruments (without plucking instruments)	18	26%		26	20%		44	22%	
Voice	8	11%		19	14%		27	13%	
Plucking instruments	9	13%		8	6%		17	8%	
Percussion instruments	6	9%		4	3%		10	5%	
Theoretical programs (composition, music theory)	1	1%		6	5%		7	4%	

*n* = 205; CI = confidence interval; differences between performance majors and music education majors assessed using chi^2^-tests and ANOVA adjusted for cohort: ^***^p ≤ 0.001. Gender: *χ*^2^(1, *n* = 203) = 14.49, *φ* = −0.27, *p* ≤ 0.001; first citizenship: *χ*^2^(1, *n* = 204) = 14.24, *φ* = −0.26, *p* ≤ 0.001; main instrument and age: n.s.

Regarding the subsample of students who completed the survey at the beginning of their first semester as well as the follow-up survey at the end of their second semester (*n* = 62), the respondents were on average 20.47 years old, 66% were female, 66% were music education majors, while 34% were performance majors, and 81% of the respondents had a German first citizenship. Regarding their main instruments, 32% of the students played keyboard instruments, 31% played wind instruments, 16% played string instruments, 11% sang, 5% played plucked instruments, 3% played percussion instruments, and 2% was enrolled in composition or music theory. A comparison of the sample characteristics of the subsample of respondents from the cohorts of 2017 and 2018 at the beginning of their first semester as well as those who completed both surveys at the beginning of their first semester and at end of their second semester is provided in Table 2.

**Table 2:** Comparison of the sample characteristics of the subsample of respondents from the cohorts of 2017 and 2018.

	Survey					
Beginning of first semester (*n* = 161)			End of second semester (*n* = 62)		
	n/M	%/SD	95% CI	n/M	%/SD	95% CI
Cohort						
2017	75	47%		27	44%	
2018	86	53%		35	57%	
Gender						
Female	84	52%		41	66%	
Male	76	47%		21	34%	
Age	20.04	2.59	[19.63, 20.44]	20.47	2.83	[19.75, 21.19]
Major						
Performance major	59	37%		21	34%	
Music education major	102	63%		41	66%	
First citizenship						
German	131	81%		50	81%	
Other	30	19%		12	19%	
Main instrument						
Wind instruments	41	26%		19	31%	
Keyboard instruments	38	24%		20	32%	
String instruments (without plucking instruments)	29	18%		10	16%	
Voice	22	14%		7	11%	
Plucking instruments	14	9%		3	5%	
Percussion instruments	10	6%		2	3%	
Theoretical programs (composition, music theory)	6	4%		1	2%	

*CI = confidence interval*.

### Health Status, Health-Related Attitudes, Knowledge, Skills and Behaviors, and Coping Strategies of Music Students at the Beginning of Their Education

The descriptive results were used to answer the first research question focusing on physical and mental health status and different health-related aspects as well as on coping strategies of music students at the beginning of their education at a music university (see Table 3). Students assessed their overall health status mainly as good (*M* = 4.10), with 55% (*n* = 112) reporting being in good and 28% (*n* = 58) even indicating being in very good health.

**Table 3:** Health status, health-related attitudes, knowledge, skills and behaviors, and coping strategies of first-year music students (performance majors and music education majors).

	Major				Total (*n* = 205)
Performance (*n* = 71, 35%)		Music education (*n* = 134, 65%)	
	M (SD; min.-max.)	95% CI	M (SD; min.-max.)	95% CI	M (SD; min.-max.)	95% CI
Health status						
Self-assessed health status	4.10 (0.64; 3–5)	[3.95, 4.25]	4.10 (0.73; 2–5)	[3.97, 4.22]	4.10 (0.70; 2–5)	[4.00, 4.19]
Pain (frequency and intensity)						
Back/shoulders	12.92 (11.0; 0–40)	[10.29, 15.55]	14.72 (13.00; 0–48)	[12.50, 16.94]	14.10 (12.36; 0–48)	[12.40, 15.81]
Arms/hands	8.16 (10.40; 0–42)	[5.68, 10.64]	6.45 (8.38; 0–45)	[5.00, 7.89]	7.04 (9.15; 0–45)	[5.77, 8.31]
Mouth/jaw	4.92 (10.40; 0–42)	[2.71, 7.12]	3.69 (8.25; 0–50)	[2.27, 5.11]	4.11 (8.60; 0–50)	[2.92, 5.31]
Hearing	2.34 (6.15; 0–35)	[0.86, 3.81]	2.00 (5.63; 0–43)	[1.03, 2.96]	2.11 (5.80; 0–43)	[1.31, 2.92]
Playing-related impairments due to pain	2.83 (1.03; 1–5)	[2.58, 3.09]	2.62 (0.99; 1–5)	[2.45, 2.79]	2.69 (1.01; 1–5)	[2.55, 2.84]
Mental health status	2.14 (0.74; 1–4)	[1.96, 2.32]	2.37 (0.89; 1–5)	[2.21, 2.52]	2.29 (0.85; 1–5)	[2.17, 2.41]
Performance anxiety	2.89 (1.07; 1–5)	[2.64, 3.15]	3.24 (1.03; 1–5)	[3.07, 3.42]	3.12 (1.05; 1–5)	[2.98, 3.27]
Health-related attitudes						
Health consciousness	4.04 (0.53; 3–5)	[3.92, 4.17]	3.94 (0.54; 2–5)	[3.85, 4.04]	3.98 (0.54; 2–5)	[3.90, 4.05]
Importance of health overall	4.49 (0.65; 3–5)	[4.34, 4.65]	4.43 (0.74; 2–5)	[4.30, 4.55]	4.45 (0.71; 2–5)	[4.35, 4.55]
Importance of health for musicians	4.62 (0.70; 2–5)	[4.45, 4.79]	4.71 (0.53; 3–5)	[4.62, 4.80]	4.68 (0.60; 2–5)	[4.60, 4.76]
Self-assessed health-related knowledge and skills						
Knowledge health risks for musicians^***^	3.24 (0.96; 1–5)	[3.02, 3.47]	2.68 (0.88; 1–5)	[2.53, 2.83]	2.87 (0.94; 1–5)	[2.74, 3.00]
Knowledge health protective measures for musicians^**^	2.96 (1.03; 1–5)	[2.71, 3.20]	2.51 (0.85; 1–5)	[2.37, 2.66]	2.67 (0.94; 1–5)	[2.54, 2.80]
Knowledge and skills regarding different aspects of musicians‘ health	3.07 (0.62; 1–5)	[2.92, 3.22]	2.99 (0.56; 1–4)	[2.99, 3.09]	3.02 (0.58; 1–5)	[2.94, 3.10]
Health-related behaviors						
Average daily practicing hours^***^	3.69 (1.47; 1–8)	[3.34, 4.05]	2.37 (1.05; 1–6)	[2.19, 2.55]	2.82 (1.36; 1–8)	[2.63, 3.01]
Coping strategies						
Coping with performance anxiety^*^	3.96 (0.86; 1–5)	[3.75, 4.16]	3.53 (0.91; 1–5)	[3.37, 3.68]	3.67 (0.91; 1–5)	[3.55, 3.80]
Social support as coping strategy	3.79 (0.94; 1–5)	[3.56, 4.01]	3.89 (0.83; 1–5)	[3.83, 4.12]	3.91 (0.87; 1–5)	[3.79, 4.03]
Positive thinking as coping strategy	3.36 (0.74; 2–5)	[3.18, 3.53]	3.15 (0.96; 1–5)	[2.99, 3.32]	3.22 (0.90; 1–5)	[3.10, 3.35]
Active coping as coping strategy	2.93 (0.85; 1–5)	[2.73, 3.13]	2.99 (0.85; 1–5)	[2.85, 3.14]	2.97 (0.85; 1–5)	[2.86, 3.09]
Faith as coping strategy	2.11 (1.00; 1–5)	[1.87, 2.35]	2.27 (1.18; 1–5)	[2.07, 2.47]	2.22 (1.12; 1–5)	[2.06, 2.37]
Alcohol and cigarettes as coping strategy	1.95 (1.09; 1–5)	[1.69, 2.21]	1.60 (0.84; 1–5)	[1.45, 1.74]	1.72 (0.95; 1–5)	[1.59, 1.85]

*n* = 205 (n_performance major_ = 71; n_music education major_ = 134); CI = confidence interval; differences between performance majors and music education majors assessed using ANOVAs adjusted for instrument group, cohort, gender, and first citizenship: ^***^p ≤ 0.001, ^**^p ≤ 0.01, ^*^p ≤ 0.05. Knowledge level health risks for musicians: F(1,181) = 6.72, *p* ≤ 0.01, $\eta_{p}^{2}$ *= 0.04; knowledge level health protective measures for musicians: F(1,182) = 5.93, *p* ≤ 0.05*, $\eta_{p}^{2}$ *= 0.03; average daily practicing hours: F(1,178) = 16.96, *p* ≤ 0.001*, $\eta_{p}^{2}$ *= 0.09; coping with performance anxiety: F(1,181) = 9.19, *p* ≤ 0.01*, $\eta_{p}^{2}$ *= 0.05; others: n.s*.

**Figure 1:**
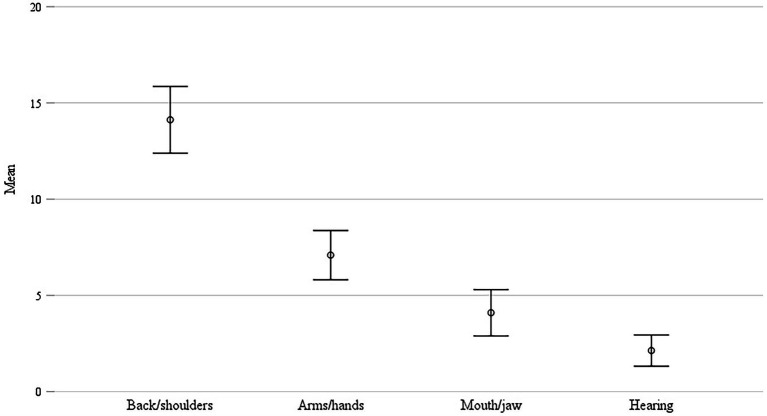
Mean of the pain score (function of frequency and severity of pain; range 0–50) in the respective body parts (*n* = 205; error bars represent 95% CI).

Regarding pain in different body regions, respondents reported the highest mean pain score in back and shoulders (*M* = 14.10, possible range of pain scores: 0 to 50), followed by arms and hands (*M* = 7.04). This suggests the back and shoulder region as the most critical body region for first-year students, with 30% (*n* = 62) reporting having pain in back and/or shoulders more than once a month and 20% (*n* = 40) even indicated suffering from permanent pain in the back/shoulder area (see Figure 1). On average, the respondents showed medium scores with regards to the playing-related impairments they feel due to pain (*M* = 2.83).

On average, the respondents reported a good mental health status (*M* = 2.29, with lower values indicating better mental health status). However, the score for performance anxiety was slightly higher (*M* = 3.12).

On average, the respondents reported to be rather health conscious (*M* = 3.98) and ascribed high importance to health overall (*M* = 4.45) and especially to health for musicians (*M* = 4.68).

Concerning health-related knowledge and skills, respondents reported medium levels of knowledge regarding health risks (*M* = 2.87) and health protective measures for musicians (*M* = 2.67), and medium levels of knowledge and skills regarding different aspects of musicians’ health (*M* = 3.02).

As an aspect of studying music which can influence the health status, we looked at the daily practicing time: On average, the respondents reported practicing 2.82 h daily (see Table 3).

Coping mechanisms are relevant to handle stressful times and there are various strategies to rely on during difficult situations. On average, the respondents reported to rely on social support as a coping strategy (*M* = 3.91), to a lower degree on positive thinking (*M* = 3.22), and active coping (*M* = 2.97). Faith (*M* = 2.22) as well as alcohol and cigarettes (*M* = 1.72) as coping mechanisms reach lower scores. Further, we assessed the respondents’ perceptions of their ability to cope with performance anxiety, with results showing rather high self-assessed abilities (*M* = 3.67).

### Differences Between Performance Majors and Music Education Majors

Since the study requirements for students enrolled in performance classes and those in music education training differ, we assumed differences between these two groups regarding the measures under investigation (H1; see Table 3).

Concerning sample characteristics, students enrolled in performance training differed from students in music education training regarding their gender, with performance majors having a higher rate of male students, and their first citizenship, with performance majors having a higher rate of students with another first citizenship than German (see Table 1). Therefore, gender and first citizenship were controlled in all ANOVAs to assess the differences between majors and instrument groups.

We found significant differences regarding the students’ self-assessed knowledge about health risks [*F*(1,181) = 6.72, *p* ≤ 0.01, $\eta_{p}^{2}$ *= 0.04] and health protective measures for musicians [*F*(1,182) = 5.93, *p* ≤ 0.05*, $\eta_{p}^{2}$ = 0.03]: Students enrolled in performance programs reported better self-assessed knowledge in both domains (health risks: *M* = 3.24; protective measures: *M* = 2.96) than their fellow students in music education programs (health risks: *M* = 2.68; protective measures: *M* = 2.51). Additionally, there were significant differences regarding the average daily practicing hours [*F*(1,178) = 16.96, *p* ≤ 0.001, $\eta_{p}^{2}$ = 0.09]: While performance majors on average practiced about 3.69 h per day, music education majors practiced on average 2.37 h daily. Finally, performance majors and music education majors also differed significantly regarding their abilities to cope with performance anxiety [*F*(1,181) = 9.19, *p* ≤ 0.01, $\eta_{p}^{2}$ = 0.05], with performance majors (*M* = 3.96) reporting better coping abilities than music education majors (*M* = 3.53).

There were no statistically significant differences between performance majors and music education majors regarding the other variables under investigation. Accordingly, the first hypothesis was only partially supported.

### Differences Between Instrument Groups

Besides differences between students enrolled in performance or in music education training, we also assumed differences between students playing different main instruments regarding the aspects under investigation (H2). Regarding sample characteristics, there were no significant differences between students playing different main instruments regarding field of study, first citizenship, and age, but regarding gender [*χ*^2^(6, *n* = 200) = 28.93, Cramer’s *V* = 0.38, *p* ≤ 0.001], with voice having the highest share of female students (*n* = 21, 78%), followed by string instruments (*n* = 32, 76%), and wind instruments (*n* = 28, 55%), while percussion instruments had the highest share of male students (*n* = 9, 90%), followed by theoretical programs without main instrument (*n* = 5, 71%), plucking instruments (*n* = 12, 71%), and keyboard instruments (*n* = 24, 52%).

Students with different main instruments differed significantly regarding their average daily practicing hours [*F*(6,178) = 3.24, *p* ≤ 0.05, $\eta_{p}^{2}$ = 0.10]. A Bonferroni-corrected post-hoc test showed that students studying theoretical programs without main instruments practiced less (*M* = 1.33, *SD* = 0.52) than students playing string instruments (*M* = 3.36, *SD* = 1.58, *p* ≤ 0.01), students playing percussion instruments (*M* = 3.30, *SD* = 1.57, *p* ≤ 0.05), and students playing keyboard instruments (*M* = 2.84, *SD* = 1.33, *p* ≤ 0.01).

Moreover, back/shoulder pain [*F*(6,181) = 2.34, *p* ≤ 0.05, $\eta_{p}^{2}$ = 0.07] and pain in the mouth/jaw [*F*(6,179) = 3.16, *p* ≤ 0.01, $\eta_{p}^{2}$ = 0.10] differed significantly between the instrument groups. A Bonferroni post-hoc analysis revealed that students playing string instruments (*M* = 19.65, *SD* = 1.58) reported significantly more frequent and intense back/shoulder pain than students who sang (*M* = 9.23, *SD* = 10.27, *p* ≤ 0.05) and students playing wind instruments reported significantly greater pain in the mouth/jaw (*M* = 8.05, *SD* = 10.89) than students playing keyboard instruments (*M* = 2.40, *SD* = 8.60, *p* ≤ 0.05). There were no statistically significant differences between students playing different main instruments regarding the other variables under investigation. Like the first hypothesis, the second hypothesis was only partially supported.

### Development of the Aspects Under Investigation Over the Course of the First Two Semesters at a Music University

To answer the second research question, we assessed differences of the subsample of first-year music students from the cohorts of 2017 and 2018 that completed the survey both at the beginning of their first semester and at the end of their second semester. We analyzed changes in the aspects under investigation over the course of their first two semesters at a music university using repeated measures ANOVAs (see Table 4).

**Table 4:** Development of health status, health-related attitudes, knowledge, skills and behaviors, and coping strategies over the course of the first two semesters at a music university (students attending and not attending courses on musicians’ health).

	Total				Attending courses (*n* = 20, 32%)				Not attending courses (*n* = 42, 68%)			
Beginning of first semester		End of second semester		Beginning of first semester		End of second semester		Beginning of first semester		End of second semester	
	M (SD)	95% CI	M (SD)	95% CI	M (SD)	95% CI	M (SD)	95% CI	M (SD)	95% CI	M (SD)	95% CI
Health status												
Self-assessed health status	3.98 (0.72)	[3.80, 4.17]	4.00 (0.77)	[3.80, 4.20]	4.05 (0.69)	[3.73, 4.37]	4.15 (0.75)	[3.80, 4.50]	3.95 (0.74)	[3.72, 4.18]	3.93 (0.79)	[3.68, 4.18]
Pain (frequency and intensity)												
Back/shoulders	15.20 (12.86)	[11.94, 18.47]	14.61 (11.40)	[11.72, 17.51]	12.00 (11.84)	[6.45, 17.54]	10.27 (9.41)	[5.86, 14.67]	16.73 (13.18)	[12.62, 20.84]	16.68 (11.77)	[13.02, 20.35]
Arms/hands	7.25 (8.68)	[5.01, 9.49]	7.73 (9.74)	[5.26, 10.20]	8.17 (10.10)	[3.44, 12.89]	7.60 (9.02)	[3.38, 11.82]	6.80 (7.98)	[4.24, 9.35]	7.79 (10.17)	[4.62, 10.96]
Mouth/jaw	6.15 (12.26)	[3.01, 9.29]	5.17 (8.81)	[2.94, 7.41]	3.72 (8.58)	[−0.30, 7.74]	2.34 (5.83)	[−0.38, 5.07]	7.33 (13.64)	[3.03, 11.64	6.52 (9.68)	[3.50, 9.54]
Hearing	3.20 (8.04)	[1.14, 5.26]	3.18 (7.12)	[1.38, 4.99]	3.81 (7.77)	[0,17, 7.45]	4.01 (6.23)	[1.06, 6.95]	2.90 (8.24)	[0.30, 5.50]	2.79 (7.52)	[0.45, 5.14]
Playing-related impairments due to pain	2.75 (0.82)	[2.53, 2.96]	2.78 (0.94)	[2.54, 3.03]	2.84 (0.83)	[2.44, 3.24]	2.90 (0.79)	[2.53, 3.27]	2.70 (0.82)	[2.44, 2.96]	2.73 (1.01)	[2.40, 3.05]
Mental health status^**^	2.28 (0.90)	[2.04, 2.51]	2.61 (0.82)	[2.40, 2.82]	2.11 (0.71)	[1.77, 2.45]	2.18 (0.71)	[1.85, 2.51]	2.35 (0.97)	[2.05, 2.66]	2.82 (0.80)	[2.57, 3.07]
Performance anxiety	3.22 (0.99)	[2.96, 3.47]	3.02 (0.98)	[2.77, 3.27]	2.88 (0.95)	[2.44, 3.33]	2.65 (0.88)	[2.24, 3.06]	3.38 (0.99)	[3.07, 3.69]	3.20 (0.98)	[2.89, 3.50]
Health-related attitudes												
Health consciousness	3.99 (0.49)	[3.87, 4.11]	4.01 (0.58)	[3.86, 4.16]	4.07 (0.50)	[3.84, 4.30]	4.01 (0.61)	[3.73, 4.29]	3.95 (0.48)	[3.80, 4.10]	4.01 (0.58)	[3.83, 4.19]
Importance of health overall	4.45 (0.72)	[4.27, 4.63]	4.55 (0.62)	[4.39, 4.71]	4.50 (0.69)	[4.18, 4.82]	4.55 (0.51)	[4.31, 4.79]	4.43 (0.74)	[4.20, 4.66]	4.55 (0.67)	[4.34, 4.76]
Importance of health for musicians	4.63 (0.63)	[4.47, 4.79]	4.68 (0.72)	[4.49, 4.86]	4.50 (0.83)	[4.11, 4.89]	4.65 (0.93)	[4.21, 5.09]	4.69 (0.52)	[4.53, 4.85]	4.69 (0.60)	[4.50, 4.77]
Health-related knowledge and skills												
Knowledge health risks for musicians	2.89 (0.89)	[2.66, 3.11]	3.02 (0.93)	[2.78, 3.25]	3.30 (0.87)	[2.90, 3.70]	3.80 (0.83)	[3.41, 4.19]	2.69 (0.84)	[2.43, 2.95]	2.64 (0.73)	[2.42, 2.87]
Knowledge health protective measures for musicians^**^	2.71 (0.95)	[2.47, 2.95]	3.05 (0.97)	[2.80, 3.29]	3.10 (0.91)	[2.67, 3.53]	3.80 (0.83)	[3.41, 4.19]	2.52 (0.92)	[2.24, 2.81]	2.69 (0.81)	[2.44, 2.94]
Knowledge and skills regarding different aspects of musicians‘ health^*^	2.97 (0.55)	[2.83, 3.11]	3.16 (0.50)	[3.03, 3.28]	2.94 (0.68)	[2.63, 3.26]	3.46 (0.46)	[3.25, 3.68]	2.99 (0.49)	[2.84, 3.14]	3.01 (0.45)	[2.87, 3.15]
Health-related behaviors												
Average daily practicing hours	2.77 (1.35)	[2.43, 3.12]	2.89 (1.30)	[2.56, 3.22]	3.84 (1.21)	[3.26, 4.43]	4.00 (1.05)	[3.52, 4.48]	2.29 (1.11)	[1.94, 2.63]	2.36 (1.10)	[2.03, 2.69]
Coping strategies												
Coping with performance anxiety	3.46 (0.99)	[3.21, 3.72]	3.58 (0.93)	[3.35, 3.82]	3.38 (0.96)	[2.94, 3.83]	3.70 (0.98)	[3.24, 4.16]	3.50 (1.02)	[3.18, 3.82]	3.53 (0.91)	[3.24, 3.82]
Social support as coping strategy	3.96 (0.86)	[3.74, 4.18]	3.97 (0.85)	[3.75, 4.19]	3.69 (0.99)	[3.22, 4.15]	3.64 (0.95)	[3.19, 4.08]	4.09 (0.78)	[3.85, 4.33]	4.13 (0.76)	[3.89, 4.36]
Positive thinking as coping strategy	3.17 (0.93)	[2.93, 3.40]	3.21 (0.79)	[3.01, 3.41]	3.35 (0.75)	[3.00, 3.70]	3.09 (0.59)	[2.81, 3.36]	3.08 (1.00)	[2.77, 3.39]	3.27 (0.87)	[3.00, 3.55]
Active coping as coping strategy	2.98 (0.75)	[2.79, 3.17]	2.86 (0.75)	[2.67, 3.05]	3.04 (0.83)	[2.65, 3.43]	2.90 (0.81)	[2.53, 3.28]	2.95 (0.72)	[2.73, 3.18]	2.84 (0.73)	[2.61, 3.07]
Faith as coping strategy	2.33 (1.09)	[2.05, 2.61]	2.46 (0.94)	[2.22, 2.70]	2.47 (1.24)	[1.89, 3.05]	2.48 (0.95)	[2.03, 2.92]	2.27 (1.02)	[1.95, 2.59]	2.45 (0.94)	[2.15, 2.74]
Alcohol and cigarettes as coping strategy	1.63 (0.91)	[1.40, 1.86]	1.51 (0.69)	[1.33, 1.68]	1.78 (1.09)	[1.26, 2.29]	1.46 (0.56)	[1.20, 1.73]	1.57 (0.81)	[1.31, 1.82]	1.53 (0.74)	[1.30, 1.76]

n = 62; CI = confidence interval; differences between beginning of first semester and end of second semester assessed using repeated measures ANOVAs: ^**^p ≤ 0.01, ^*^p ≤ 0.05. Mental health status: F(1.59) = 8.49, *p* ≤ 0.01, $\eta_{p}^{2}$ *= 0.13; knowledge level health protective measures for musicians: F(1.61) = 7.50, *p* ≤ 0.01*, $\eta_{p}^{2}$ *= 0.11; assessment of knowledge and skills regarding different aspects of musicians‘health F(1.61) = 4.29, *p* ≤ 0.05*, $\eta_{p}^{2}$ *= 0.07; others: n.s*.

The results showed that the students’ mental health status was significantly worse at the end of the second semester at a music university [*F*(1.59) = 8.49, *p* ≤ 0.01, $\eta_{p}^{2}$ = 0.13; *M*_beginning first semester_ = 2.28, *M*_end second semester_ = 2.60, with lower values indicating better mental health status).

However, self-assessed knowledge about health protective measures for musicians increased significantly from the beginning of the music university education to the end of the second semester [*F*(1.61) = 7.50, *p* ≤ 0.01, $\eta_{p}^{2}$ = 0.11, *M*_beginning first semester_ = 2.71, *M*_end second semester_ = 3.05] and there was also a small but significant increase between time points concerning the self-assessment of knowledge and skills regarding different aspects of musicians‘health [*F*(1.61) = 4.29, *p* ≤ 0.05, $\eta_{p}^{2}$ = 0.07; *M*_beginning first semester_ = 2.97, *M*_end second semester_ = 3.16].

There were no statistically significant differences between the beginning of the first semester and the end of the second semester regarding the other variables under investigation.

### Differences Over the Course of the First Two Semesters at a Music University Between Students Taking Courses on Musicians’ Health and Students Not Taking Courses

To test H3, we looked at the interactions between students attending or not attending courses on musicians’ health and the development of the aspects under investigation over time (group x time). Table 4 provides an overview of the mean scores of the variables under investigation for students who attended courses on musicians’ health (*n* = 20, 32%) and those who did not (*n* = 42, 68%) at the beginning of their first semester and at the end of their second semester.

**Figure 2:**
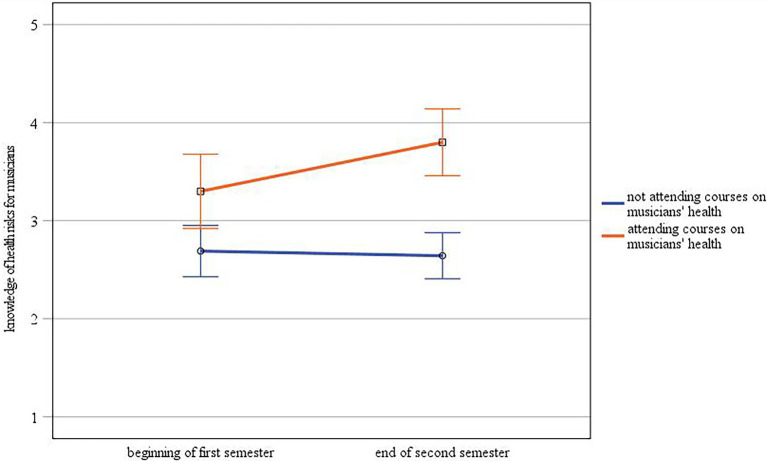
Interaction between time and group regarding self-assessment of knowledge about health risks for musicians (error bars represent 95% CI).

Regarding the self-assessed knowledge of health risks for musicians, there was a statistically significant interaction between time and group [*F*(1.60) = 4.03, *p* ≤ 0.05, $\eta_{p}^{2}$ = 0.06; see Figure 2]. A one-way ANOVA to test the main effect of group showed significant differences between students taking courses on musicians’ health and those students who did not both at the beginning [*F*(1.60) = 7.00, *p* ≤ 0.01, $\eta_{p}^{2}$ = 0.10] and at the end of the semester [*F*(1.60) = 31.24, *p* ≤ 0.001, $\eta_{p}^{2}$ = 0.34], with students having attended musicians’ health-related courses reporting higher knowledge levels at both time points (beginning of first semester: *M*_attending courses_ = 3.30, *M*_not attending courses_ = 2.69; end of second semester: *M*_attending courses_ = 3.80, *M*_not attending courses_ = 2.64). Repeated measures ANOVA to test the main effect of time showed that there was no main effect for time on knowledge of health risks for musicians neither for students taking courses [*F*(1.19) = 3.52, *p* = 0.08, $\eta_{p}^{2}$ = 0.16] nor for students not taking courses [*F*(1.41) = 0.12, *p* = 0.74, $\eta_{p}^{2}$ = 0.00].

**Figure 3:**
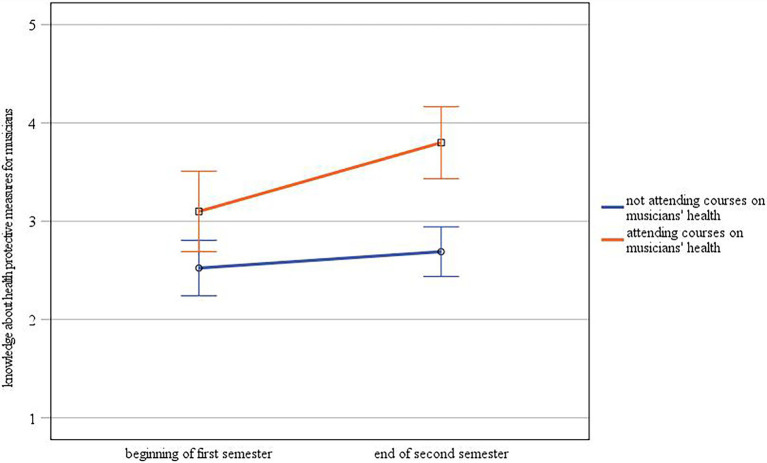
Interaction between time and group regarding self-assessment of knowledge about health protective measures for musicians (error bars represent 95% CI).

Regarding self-assessed knowledge about health protective measures for musicians, there was a statistically significant interaction between time and group [*F*(1.60) = 4.28, *p* ≤ 0.05, $\eta_{p}^{2}$ = 0.07; see Figure 3]. A one-way ANOVA showed that knowledge about health protective measures for musicians was significantly higher for students taking courses on musicians’ health than for those students who did not both at the beginning [*F*(1.60) = 5.37, *p* ≤ 0.05, $\eta_{p}^{2}$ = 0.08; *M*_attending courses_ = 3.10, *M*_not attending courses_ = 2.52] and at the end of the semester [*F*(1.60) = 24.91, *p* ≤ 0.001, $\eta_{p}^{2}$ = 0.29; *M*_attending courses_ = 3.80, *M*_not attending courses_ = 2.69]. As reported above, we found a significant difference between time points. A further repeated measures ANOVA with separate examination of the two groups revealed that there was a statistically significant effect of time on knowledge about health protective measure only for the group of students who were taking courses on musicians’ health, [*F*(1.19) = 9.22, *p* ≤ 0.01, $\eta_{p}^{2}$ = 0.33] but not for the group who did not attend courses [*F*(1.41) = 1.41, *p* = 0.24, $\eta_{p}^{2}$ = 0.03], with students attending courses on musicians health showing an increase in knowledge at the end of the second semester compared to the beginning of the first semester.

**Figure 4:**
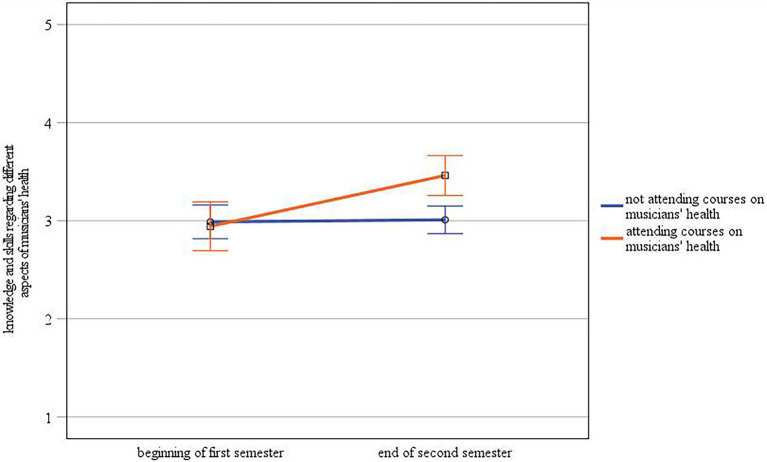
Interaction between time and group concerning self-assessment of knowledge and skills regarding different aspects of musicians’ health (error bars represent 95% CI).

Finally, there was a statistically significant interaction between time and group concerning the self-assessment of knowledge and skills regarding different aspects of musicians’ health [*F*(1.60) = 7.85, *p* ≤ 0.01, $\eta_{p}^{2}$ = 0.12; see Figure 4]. A one-way ANOVA showed that the assessment of knowledge and skills regarding different aspects of musicians’ health differed significantly between students who took courses on musicians’ health and those students who did not at the end of the second semester [*F*(1.60) = 13.39, *p* ≤ 0.001, $\eta_{p}^{2}$ = 0.18] but not at the beginning of the first year [*F*(1.60) = 0.09, *p* = 0.77, $\eta_{p}^{2}$ = 0.00]. In the analysis reported above, we found significant differences between time points. A repeated measures ANOVA with separate examination of the two groups further revealed a statistically significant simple main effect of time on knowledge and skills regarding different aspects of musicians’ health for the group of students who were attending courses on musicians’ health [*F*(1.19) = 7.50, *p* ≤ 0.05, $\eta_{p}^{2}$ = 0.28], who assessed their knowledge and skills as higher at the end of their second semester (*M* = 3.46) compared to the beginning of their first semester (*M* = 2.94), but not for the group who did not attend such courses [*F*(1.41) = 0.06, *p* = 0.80, $\eta_{p}^{2}$ = 0.00; *M*_beginning first semester_ = 2.99, *M*_end second semester_ = 3.01].

There were no statistically significant interaction effects regarding the other variables under investigation. Accordingly, the third hypothesis could only be supported with regard to health-related knowledge and skills, but not with regard to health status and health-related attitudes.

## Discussion

Health is an important issue for music students. To address their specific needs, it is essential to know their health status, health-relevant attitudes and behaviors when they first enter a music university. Therefore, the aim of our study was to examine the physical and mental health status of first-year music students, their health-related attitudes, knowledge, skills, and behaviors, and their coping strategies. In doing so, we also investigated differences between performance and music education majors as well as differences between students playing different main instruments. Additionally, we aimed to analyze how these aspects under investigation changed during the first two semesters at a music university and whether there were differences over time between students who attended courses on musicians’ health and students who did not attend such courses.

Concerning H1, unexpectedly our study revealed little differences in health status and health-related attitudes between performance majors and music education majors. Although music performance majors practiced more than one hour more per day as compared to music education majors, both groups show a similar occurrence of pain syndromes and performance anxiety. However, compared to the large studies of Spahn et al. (2004), Kreutz et al. (2009), and Brandfonbrener (2009), the students assessed their overall health status and their mental health status at the beginning of their first semester better and mainly as good.

In line with previous research (Williamon and Thompson, 2006), our results show that the back and shoulder region is the most critical body region for first-year students regarding pain. This is especially true for students playing string instruments, which is in line with studies showing that professional string players experience physical problems more frequently (Gembris et al., 2018). Students playing violin and viola have ergonomically greater physical strains due to the playing posture as compared to other instrumentalists (Steinmetz et al., 2015). As one would expect, our results further showed a trend that students playing wind instruments suffered more from pain in the mouth/jaw than students playing other main instruments. However, regarding H2, differences in health status between students playing different main instruments were not significant.

As reported in previous findings (e.g., Perkins et al., 2017), music students seem to know the importance of health for musicians and reported an increased health consciousness. However, their perceived knowledge regarding health risks and health protective measures was a little less pronounced but still on a medium level. Students enrolled in performance programs self-assessed their knowledge about health risks and protective behaviors as better than their fellow students enrolled in education training. This difference underlines that performance majors, who also practiced on average about 1,5 h more per day, may have already encountered health problems and are, thus, more aware of the risks for musicians (Gembris et al., 2020). Furthermore, their peers might more frequently address preventive measures, warm-ups, and practice techniques. On the one hand, these findings underline the concern students show about their health from an early point in their education on, on the other hand, these findings point to the importance of considering different needs of different majors when teaching music students techniques and measures to maintain their health. It should be mentioned, that 30% of adolescent high performing musicians suffering from injury or playing-related pain feel not taken seriously by their instrumental teachers (Gembris et al., 2020). Concerning university students, our assumption that performance majors and music education majors differ regarding the aspects under investigation was only supported regarding self-assessed knowledge about health risks and health protective measures for musicians, the average daily practicing hours, and abilities to cope with performance anxiety. With regard to the latter, it was shown that performance majors assessed their abilities to cope with performance anxiety as better than music education majors. Here, probably a selection bias has to be assumed, since students suffering from performance anxiety tend to display avoidance behavior and might chose programs implying fewer public appearances (Schneider and Chesky, 2011). This can be remediated by early interventions in music schools targeted at overcoming music performance anxiety already early in the career (Braden et al., 2015).

As a positive outcome, and in contrast to previous research showing music students’ poor use of coping strategies (Araújo et al., 2017), the first-year students in our sample reported healthy coping strategies, with social support and positive thinking as the most used strategies. Concerning RQ2, the results are overall in line with previous research: Comparable to Zander et al. (2010), we found a decrease in music students’ mental health status over the course of the first two semesters at a music university. Students still reported medium mental health scores at the end of their second semester, however, a worsened mental health status is concerning. Decline in mental health status might also be due to the changes involved in starting studying at a university, often accompanied by leaving the childhood home – often the hometown or even country – and having to become more independent. But since this result is in line with studies showing that music students particularly suffer from mental distress (e.g., Wristen, 2013), the decrease in mental health status might also be due to musician specific factors such as high demands, high ambitions, high level of competition and specific stressors linked to adaptation to new teachers, and new practice habits. In line with this, Hildebrandt et al. (2012) found an increase of fatigue, depression, and stage fright during the first year of high-level education in a Swiss music university.

Regarding H3, the results concerning the effects of attending the comprehensive health program were disappointing. The small sample of 20 students taking the courses can be explained by the fact that students are free to attend these obligatory courses at any semester of their education, and, thus, seem to postpone them to a later timepoint, after the second semester. This has two reasons: first, in their first two semesters, students want to concentrate on improving their instrumental skills since they are afraid to disappoint their teachers. Second, non-German-speaking students tend to choose these courses at a later timepoint, when they have improved their language skills.

Comparing the development of the aspects under investigation in the 20 students having attended courses on musicians’ health in their first two semesters to the 42 students having not attended, our results point toward a potentially health-enhancing impact of such courses. The findings showed that self-assessed knowledge about health protective measures for musicians as well as self-assessed knowledge and skills regarding different aspects of musicians’ health was significantly better at the end of the second semester compared to the beginning of the first semester for those students attending courses on musicians’ health. However, neither pain, playing-related impairments due to pain nor general or mental health status or performance anxiety was improved at the end of the second semester in course taking students. A possible explanation could be, that students at risk may have chosen the courses, and thus prevented a deterioration of their health status. Generally, selection biases of such courses among those who are already sensitized play a role here and are described also by other researchers when drawing non-randomized samples (e.g., Spahn et al., 2004). In any case, studies under controlled conditions are necessary to investigate the causal effects of such courses; especially studies examining health-related attitudes, for which we found no differences between students who attended courses on musicians’ health and those who did not. But since attitudes are important predictors of actual behavior, changing attitudes that may then translate into behavior is also a relevant outcome measure of such courses (e.g., Link et al., 2021). Additionally, future studies should consider further time points to explore long-term effects of courses on musicians’ health.

Our findings also showed that students attending courses on musicians’ health assessed their knowledge of health risks and health protective measures for musicians as better than students not attending such courses already at the beginning of their first semester. This result suggests students being more aware of health threats for musicians are more willing to take courses on musicians’ health early on in their education, which points to the need to address students in their first semester and raise their awareness regarding such topics. This has been also emphasized in a similar, 6 months follow-up study evaluating a health education program in beginner music students (Matei et al., 2018).

As a side note, health status and health behaviors at our university seem to have improved during the last 18 years. We distributed a similar questionnaire in 2002 to 340 bachelor and master students. The return rate was 62% (*n* = 217). Questions concerning playing-related pain location and pain frequency resulted in quite dramatic percentages: 40% of students reported constant (playing-related) shoulder pain and 37% reported constant playing-related back-pain. Since multiple responses were allowed, altogether more than 60% reported playing-related shoulder or back-pain (Gräser, 2004). Since this number is three times higher than in the present study, it may be indicative of an improvement of general health status and health behavior in our music students. However, it has to be taken into consideration that we used different wordings for the questionnaire and we included all bachelor and master students, not only first years. However, as a consequence of these results, we implemented a health program, specified in the introduction (Altenmüller, 2014). Furthermore, we regularly addressed health issues in public and private music schools and implemented regular training aiming to inform music teachers on “healthy music making” (Schuppert and Altenmüller, 2016).

Our study has several limitations that should be considered when interpreting the results. First, several measures were assessed with single items. Future studies should consider the aspects under investigation more comprehensively. Second, all results are based on participants’ self-assessments and are, thus, subjective. Future studies should combine self-reported measures with objective observational data. Third, compared to music education majors, performance majors were underrepresented in the sample. This is probably due to the fact that our international students, who amount to about 60% of the performance majors, are frequently reluctant to fill in questionnaires in German or English language. In contrast, music education majors are mostly German-speaking (about 80%). Fourth, a possible selection bias should be considered since students participating in the survey might differ significantly from those who did not participate. Fifth, due to the data collection at only one music university and the small sample size, the generalizability of the results remains questionable. Sixth, the assessment of differences between students attending courses on musicians’ health and those who did not were based on small groups and the assignment to one of the two groups was not randomized since the students could take such courses in their first two semesters at their own discretion. Finally, students were not surveyed after a full year at the university, but at the end of their second semester. Accordingly, the results at the second time point could be influenced by the potentially stressful phase at the end of a semester.

## Conclusion and Practical Suggestions for Programs Addressing Health Issues for Music Students

Overall, our results provide some insights into the bodily and mental health status, health-related attitudes, behaviors, knowledge, and skills as well as coping strategies of music students at the beginning of their music university education in our specific institution. Generally, health status and health-related attitudes seem to have improved over the last decades, however, direct comparison to other studies remains difficult, since questionnaires applied and wordings of the questions differ in the above mentioned studies. Furthermore, health attitudes and well-being are dependent on many bio-psycho-social determinants, including music- and study-related factors, such as study organization, workload, minor subject, percentage of international students, but also on bio-societal and socio-ecological factors including environment, nutrition behavior, work ethics etc. (for an overview concerning differences between music students and students from nursing or biomedical sciences see Ginsborg et al., 2009). Generally, an almost infinite number of biological (partially innate), socio-economic and other societal factors, many of them dynamically changing, determine health behaviors, which has been exemplified in adolescents’ mental health in a recent review by Currie and Morgan (2020).

Further, this study offers some novel insights about the development of health-related aspects over the course of the first two semesters at a music university and the possible impact of courses on musicians’ health, whereby selection bias may have influenced our results. Disappointingly, taking health-related courses does not improve music students’ health, however, not astonishingly, informs students about health behaviors.

Five key points can be derived from the above:

1. Knowledge about music students’ specific health challenges at the beginning of their university education can help music universities to better respond to the needs of their students and inform future measures to help music students maintain healthy over the course of their university education.

2. Generally, health status and knowledge about health-related behaviors in first-year music students are not satisfactory. Here, information and habit building needs to start earlier: in music schools and high schools. Peers and teachers have a pivotal role in the transmission of knowledge.

3. Music universities need to respond to the increasing challenges of cultural economy. Students have to be empowered to cope with the many stressors they will meet during their professional life. Therefore, it is extremely important, to draw music students’ attention to health-related topics, to change their health-related attitudes, to raise awareness, and to teach them adequate measures to preserve good health. This seems especially important since professional musicians show poor health behaviors (Kenny et al., 2014).

4. Generally, this education – and even more important – habit formation should take place during the first year of study. This will raise awareness, prepare, and enable musicians later in their career to incorporate and engage in healthy behaviors.

5. Offering a comprehensive health program as we do is “nice-to-have” and students value it, however, future research should focus on appropriate measures to improve the transfer from the lecture hall to real life.

## Data Availability Statement

The raw data supporting the conclusions of this article will be made available by the authors, without undue reservation.

## Ethics Statement

The studies involving human participants were reviewed and approved by Joint Ethics Committee of the Leibniz University Hannover and the Hanover University of Music, Drama and Media (EV-LUH 9/2017). The participants provided their written informed consent to participate in this study.

## Author Contributions

MR designed and performed the study, evaluated the questionnaires, conducted the statistics, and wrote the manuscript. EB designed and performed the study, evaluated the questionnaires, and wrote the manuscript. EA designed the study, recruited the students, did the health program, evaluated the questionnaires, and wrote the manuscript. All authors contributed to the article and approved the submitted version.

## Conflict of Interest

The authors declare that the research was conducted in the absence of any commercial or financial relationships that could be construed as a potential conflict of interest.

## Publisher’s Note

All claims expressed in this article are solely those of the authors and do not necessarily represent those of their affiliated organizations, or those of the publisher, the editors and the reviewers. Any product that may be evaluated in this article, or claim that may be made by its manufacturer, is not guaranteed or endorsed by the publisher.
